# Research Progress of Additively Manufactured Metallic Lattice Structures

**DOI:** 10.3390/mi16121418

**Published:** 2025-12-17

**Authors:** Chenchen Tian, Yongjian Wang, Haiyang Fan, Xuekun Li

**Affiliations:** 1 School of Mechanical Engineering, Shandong University, Jinan 250061, China; wangyongjian530@mail.sdu.edu.cn; 2 Key Laboratory of High Efficiency and Clean Mechanical Manufacture of Ministry of Education, Shandong University, Jinan 250061, China; 3 Yantai Research Institute, Harbin Engineering University, Yantai 264000, China; 4 Department of Mechanical Engineering, Tsinghua University, Beijing 100084, China; xli@tsinghua.edu.cn

**Keywords:** additive manufacturing, metallic lattice structure, design, process, performance

## Abstract

Metallic lattice structures have emerged as a crucial carrier for structure–function integration, owing to their exceptional mechanical properties, energy absorption performance, thermal properties and biocompatibility. Due to the layer-by-layer deposition principle, additive manufacturing enables the precise digital fabrication of complex metallic lattice structures, breaking through the limitations of traditional manufacturing processes. This paper systematically reviews the research progress of additively manufactured metallic lattice structures. First, it categorizes and elaborates on the design methods of typical lattice structures. Second, it compares the core additive manufacturing processes in forming precision and efficiency for metallic lattice structure. Third, it summarizes the application advantages and practical cases of metallic lattice structures in mechanical properties, energy absorption performance, thermal properties, and biocompatibility. Finally, the paper proposes current challenges and prospects the development directions for enhancing the performance of additively manufactured metallic lattice structures.

## 1. Introduction

With the rapid development of the high-end equipment manufacturing industry, crucial fields such as aerospace, automotive industry, and medical and health care have shown an increasing tendency in their demands for materials and structures towards lightweight, high performance, and multi-functionality [1]. Metallic lattice structures are a type of multifunctional material composed of interconnected struts, plates, or three-dimensional curved surfaces as basic units, which are arranged in a repetitive and regular manner [2]. This structure demonstrates high specific strength and stiffness, along with superior heat dissipation and energy absorption capabilities, and holds great potential for realizing the integrated design of structure and function [3,4]. Metallic lattice structures possess extremely high designability, and can achieve multifunctional characteristics or lightweight and high-load-bearing properties that traditional materials cannot realize through the design of different cell structures [5,6]. However, traditional manufacturing methods are limited to producing metallic lattice structures with simple configurations and face challenges such as complex processes and high costs, which significantly hinder their engineering applications [7].

Additive Manufacturing (AM) technology, also known as 3D printing technology, is a technique that fabricates solid objects through a method of layer-by-layer material accumulation [8]. The development of additive manufacturing (AM) technology has broken the constraint that traditional manufacturing processes cannot produce complex metallic lattice structures. It enables precise digital manufacturing of complex lattice structures and, in theory, can fabricate solid parts of any shape, including those with porous structures [9].

In recent years, significant advances have been made in the research of additively manufactured metallic lattice structures, including innovations in structural design, manufacturing processes, and performance optimization. This paper aims to systematically sort out and summarize the latest advances in the research of additively manufactured metallic lattice structures, mainly including the classification, design, processes, and properties of metallic lattice structures. This article provides a more comprehensive perspective on the current state of research on additively manufactured metallic lattice structures and fills the gap in delivering an integrated and systematic description of their design, fabrication, and applications. Additionally, it analyzes the shortcomings of existing research and future development trends, so as to provide ideas and references for improving the performance of additively manufactured metallic lattice structures.

## 2. Design of Metallic Lattice Structure for Additive Manufacturing

### 2.1. Design of Truss Lattice Structure

**Figure 1:**
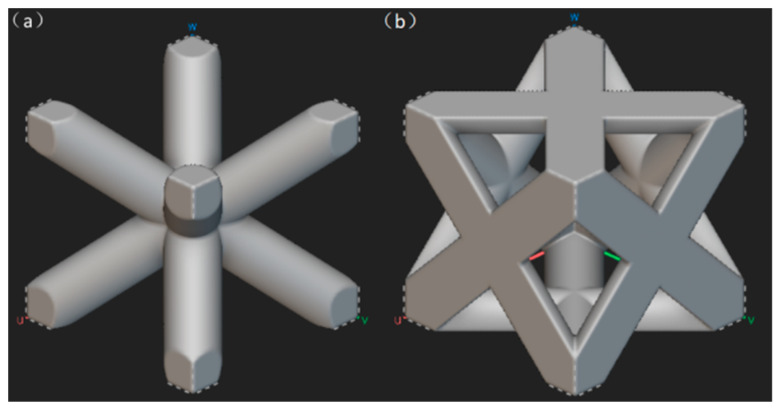
Common truss structures: (**a**) BCC truss structure; (**b**) FCC truss structure.

The unit cell of a truss lattice structure consists of struts and nodes, where specific nodes within the cell are connected at their geometric positions via struts. Variations in the truss connection modes within the unit cell exert a significant influence on mechanical properties. Truss lattice structures allow adjustment of parameters such as strut orientation and thickness within the unit cell, enabling effective control of mechanical properties, including anisotropy. By converting external loads into axial tensile and compressive stresses in the rods, they achieve efficient matching between material efficiency and mechanical perform [10]. With its outstanding mechanical properties and lightweight characteristics, the truss structure has been widely applied in fields such as architecture, bridge engineering, and aerospace. In recent years, the development and maturation of additive manufacturing (AM) technology, based on layer-by-layer deposition, has enabled the high-precision fabrication of complex truss lattice structures. As shown in Figure 1, metallic truss lattice structures represented by body-centered cubic (BCC) and face-centered cubic (FCC) configurations have become research hotspots.

In traditional truss lattice structure design, basic truss lattices are mostly used as fundamental units, which are periodically arranged to construct macroscale structures. However, this design method has limited effectiveness in optimizing structural performance; it not only hinders the full utilization of the advantages of truss lattice structures but also provides little improvement to their performance limitations. When truss lattice structures bear compressive loads, stress concentration tends to occur at their nodes, leading to premature structural failure. To optimize the performance of truss lattice structures and make up for their shortcomings, researchers have proposed a variety of novel truss structure design methods. For example, Wang et al. [11] designed a novel composite plate-truss lattice structure by combining plate structures with truss structures; this hybrid truss structure integrates the lightweight property of truss structures and the stability of plate structures. Feng et al. [12] designed a hybrid truss structure with more stable elastic modulus by combining pyramidal structures and truss structures. Hybrid truss lattice structures can effectively integrate the advantages of truss structures and other structures, significantly enhancing the performance of truss structures. In recent years, topology optimization algorithms have provided new design ideas for truss lattice structures. In the design of truss lattice structures, topology optimization algorithms can determine the optimal layout and dimensions of the rods in the truss structure, and design gradient structures to enhance the mechanical properties of the overall truss structure. For example, Jin et al. [13], based on the body-centered cubic (BCC) truss structure and using a topology optimization algorithm, designed a truss lattice structure with optimized strut diameters; the specific strength of the BCC truss structure after topology optimization design was increased by 67%. Similarly, Zhang et al. [14] designed a gradient body-centered cubic (BCC) truss lattice structure based on a topology optimization algorithm; the BCC lattice structure after topology optimization can significantly enhance stiffness and energy absorption capacity. The design of gradient truss structures based on topology optimization enables a high degree of alignment between functional requirements and mechanical properties, while preserving and improving the inherent lightweight advantage of truss lattice structures.

Truss lattice structures possess excellent designability and tunability, allowing them to be applied across a wide range of fields and to meet different performance requirements through parameter adjustments. For example, when truss structures are applied in the field of heat dissipation, it is necessary to leverage their advantage of high specific surface area. Vaissier et al. [15] adjusted the beam diameter of the truss lattice structure and the size ratio of unit cells to achieve the maximum specific surface area, thereby maximizing the cooling effect of truss structure-based heat sinks. In addition, to obtain more excellent mechanical properties, the combination of internal rods in the truss structure and their parameters can be adjusted. For example, Challapalli and Li [16] replaced solid rods with porous bionic rods, which significantly improved the energy absorption capacity of the entire truss structure.

Truss structures are also limited by their unique geometric configurations, leading to several issues. In terms of mechanical properties, most truss lattice structures (BCC) are bending-dominated. When subjected to loads, they tend to undergo bending deformation, which causes stress concentration to easily occur at the node positions where rods are connected, and subsequent failure may ensue. At the fabrication level, the rods of truss lattice structures typically have small diameters, and the spatial geometry at the nodes is complex, which places very high demands on the forming accuracy of additive manufacturing. Furthermore, the slender struts in truss lattice structures, due to their small diameters, may not receive sufficient laser or electron beam coverage across the entire cross-section. This inadequate energy input can lead to partially unmelted powder, resulting in pore formation within the struts and consequently diminishing their mechanical strength. In addition, thin inclined struts possess limited structural rigidity, making them prone to warping and localized deformation during the additive manufacturing process.

### 2.2. Design of Honeycomb Lattice Structure

**Figure 2:**
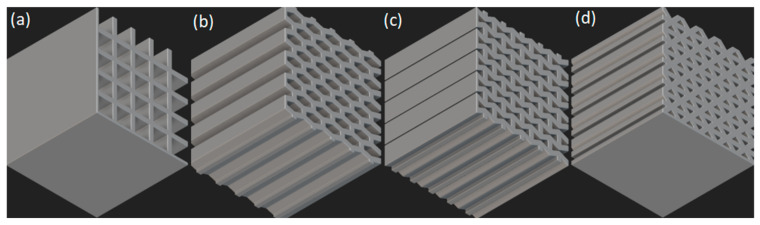
Common honeycomb structures: (**a**) square honeycomb structure; (**b**) hexagonal honeycomb structure; (**c**) re-entrant honeycomb structure; (**d**) triangular honeycomb structure.

Honeycomb lattice structures are a structural system composed of periodically arranged two-dimensional units. As shown in Figure 2, common honeycomb lattice structures exhibit excellent synergistic advantages of lightweight and mechanical properties through the ordered arrangement of planar patterns in a two-dimensional plane [17]. Honeycomb structures possess the advantage of multi-dimensional performance synergy. They exhibit characteristics of high specific stiffness and specific strength, and the load-bearing efficiency of the structure can be effectively improved by optimizing the unit cell size and arrangement [18]. Meanwhile, honeycomb structures exhibit excellent energy absorption capacity. Through structural design, they can possess negative Poisson’s ratio characteristics, which significantly enhance the energy absorption density [19]. For instance, the energy absorption density of concave honeycomb structures under high strain rate impact is increased by more than 60% compared with that of traditional regular honeycomb structures. Owing to their outstanding energy absorption capacity, honeycomb structures have ideal crashworthiness and are widely used in engineering protection, particularly in energy-absorbing components for automobile collisions [20,21].

The design of traditional honeycomb lattice structures is primarily based on regular hexagonal units, and the overall structural performance is adjusted by modifying parameters such as side length, wall thickness, and layer thickness. However, this method has a limited design space, making it difficult to achieve precise regulation and optimization of structural performance. Compared with single lattice structures, hybrid honeycomb lattice structures can simultaneously achieve multiple performance objectives—including low weight, high specific strength, and high energy absorption efficiency—through the complementary combination of different units and materials. For example, truss-honeycomb hybrid lattices not only retain the high specific stiffness of truss structures but also enhance energy absorption capacity through the buckling deformation of honeycomb units. Based on this principle, Yin et al. [22] designed a microlattice truss-reinforced honeycomb structure, and the mechanical properties and energy absorption capacity of this hybrid structure are significantly superior to those of traditional truss structures and honeycomb structures. Truss-honeycomb hybrid structures, also known as honeytube structures, are novel honeycomb structures reinforced by truss structures. Xu et al. [23] once conducted performance tests on various types of honeytube structures, and the study confirmed that honeytube structures possess excellent energy absorption capacity and compressive performance. Owing to their outstanding energy absorption capacity, truss-honeycomb hybrid structures are suitable for load-bearing components subjected to bending and impact loads. Additionally, the development of finite element technology and topology optimization has provided new approaches for honeycomb lattice structures. By establishing a finite element model of the honeycomb lattice structure, simulating its mechanical responses under different loads to obtain simulation results, and then adjusting the structural parameters based on these results, the performance of the structure can be optimized. For example, Zou et al. [24] designed a topology optimization-based inhomogeneous honeycomb structure to optimize the in-plane direction of honeycomb structures, and the mechanical properties and energy absorption capacity of this structure were significantly improved. The vulnerability of honeycomb structures in the in-plane direction under concentrated loads has attracted widespread attention. Addressing the same issue, Xing et al. [25] designed an inhomogeneous honeycomb structure by adopting a topology optimization method that maps the density to the wall thickness of honeycomb units, which significantly improved the load-bearing capacity. When traditional regular hexagonal honeycomb structures undergo in-plane compression, their straight edges and regular geometric shapes tend to cause stress concentration, resulting in poor in-plane compressive performance of the honeycomb structures. To optimize the in-plane compressive performance of honeycomb structures, most scholars choose to modify the shape of the honeycomb structure’s cell walls. For example, Feng et al. [26] found that curved-wall honeycombs can effectively alleviate the stress concentration issue of straight-wall honeycombs under in-plane compressive loads. Besides modifying the inner walls of honeycombs, some scholars also enhance in-plane compressive performance by altering the regular hexagonal configuration of traditional honeycomb structures. For instance, Ma et al. [27] designed an origami-shaped honeycomb structure, whose in-plane compressive performance and energy absorption capacity are significantly superior to those of traditional hexagonal honeycomb structures. Similarly, Montazeri et al. [28] designed two types of asymmetric star-shaped honeycomb structures to replace regular-shaped ones; their study revealed that the compressive performance of asymmetric star-shaped honeycomb structures is notably better than that of traditional honeycomb structures. In recent years, research on honeycomb lattice structures has mostly focused on developing novel honeycomb lattice structures—including irregularly shaped honeycomb structures and gradient honeycomb lattice structures—to optimize their mechanical properties. Honeycomb structures can have their properties optimized by adjusting parameters such as the shape, size, and wall thickness of honeycomb units, enabling them to meet the requirements of specific application fields.

Honeycomb lattice structures still have several issues, which mainly focus on three aspects: significant anisotropy in mechanical properties, sensitivity to manufacturing process defects, and limited operational reliability. In terms of mechanical properties, honeycomb structures, as typical lightweight porous materials, exhibit pronounced differences between their in-plane and out-of-plane properties; specifically, the compressive strength, stiffness, and overall compressive performance in the in-plane direction are much lower than those in the out-of-plane direction. In terms of manufacturing processes, as complex lattice structures, honeycomb structures are generally fabricated using additive manufacturing; however, their mechanical properties are highly sensitive to the inherent defects of additive manufacturing processes. For example, a research team led by Professor Zheng from Northwestern Polytechnical University [29] found that process defects in additive manufacturing, such as interlayer pores and inter-column pores, can cause structural failure of honeycomb structures and reduce their load-bearing capacity. Ouyang et al. [30] also found that different types of additive manufacturing defects have a significant impact on the mechanical properties of honeycomb structures. In terms of service life, the performance of honeycomb structures is easily affected by the environment and their service life is short. For instance, M. Benedetti et al. [31] found that honeycomb structures used in construction are particularly prone to fatigue damage.

The distinctive geometry of honeycomb lattice structures makes them particularly susceptible to manufacturing defects during the additive manufacturing process, thereby reducing their mechanical strength. First, the thin-walled cells of honeycomb architectures create a very narrow heat-affected region, where insufficient energy coverage and melt-pool instability can easily occur during laser or electron beam scanning. This often results in partially unmelted powder and lack-of-fusion defects, leading to the formation of interlayer pores and inter-column pores that significantly reduce the load-bearing capacity of the structure. Moreover, the high porosity and large unsupported spans inherent to honeycomb structures limit the degree of powder support and constraint during layer-wise fabrication. As the melt pool traverses these open cells, it is prone to shrinkage, sagging, or even rupture, ultimately giving rise to geometric imperfections such as distortion, collapse, and non-uniform wall thickness. Collectively, these additive manufacturing defects can severely degrade the mechanical performance of honeycomb lattice components.

### 2.3. Design of Surface Lattice Structure

The minimal surface theory provides an important mathematical foundation for the design of surface lattice structures. Smooth surfaces with periodic symmetry are obtained by solving implicit function equations, and surface lattice unit cells are constructed accordingly—this has made triply periodic minimal surfaces (TPMS) attract significant attention. As shown in Table 1 are common TPMS structures and their corresponding implicit function equations. Triply periodic minimal surfaces (TPMSs) are composed of spatially continuous zero-curvature surfaces, thus enabling the realization of lattice structures with rich morphologies, smooth transitions, and interconnected features [32,33]. This topological feature enables it to reduce stress concentration inside the structure when subjected to compressive loads, improve the stability of structural deformation, and enhance the yield strength and energy absorption characteristics of the structure [34]. TPMS structures are widely applied across various fields due to their advantages such as high specific surface area, excellent interconnectivity, remarkable energy absorption capacity, as well as superior thermal conductivity, permeability, and mechanical properties. Triply periodic minimal surface lattice structures generally exhibit superior mechanical properties, including stiffness and strength, compared to truss lattice structures of comparable mass, demonstrating significant potential particularly in energy absorption applications [35,36].

Triply periodic minimal surfaces (TPMSs) are controlled by implicit function equations. By adjusting the parameters of the implicit functions, the type and size of TPMS lattices can be modified, thereby enabling the control of structural performance. This design method can effectively avoid the stress concentration phenomenon commonly found in traditional lattice structures and make the load distribution of lattice structures more uniform. However, due to the limitations of implicit function equations, the types of TPMS lattices that can be generated are fixed, and their performance advantages differ significantly. To combine the advantages of multiple TPMS lattice types, many scholars have integrated various TPMS lattice types into a single structure. For example, Zhao et al. [37] integrated two types of TPMS structures with advantageous properties, which significantly improved the overall mechanical performance. Similarly, Gao et al. [38] periodically mixed two types of TPMS structures; the resulting hybrid TPMS structure exhibits broader and more flexible customizability, with its performance significantly optimized. Additionally, in the design of surface lattice structures, topology optimization can be used to determine the optimal type and layout of unit cells. For instance, Wang et al. [39] designed an adaptive gradient TPMS structure based on the stress field obtained from ABAQUS compression simulations, and the compressive performance of this structure was significantly enhanced. For different types of TPMS unit cells, their mechanical properties vary remarkably; during the design process, topology optimization can help select the optimal unit cell type. For example, El Khadiri et al. [40] derived the lattice structure most suitable for additive manufacturing based on topology optimization results. Beyond the selection of unit cells, topology optimization results can also be used to design the macro-layout and gradient of the final structure. For example, Strömberg [41] adopted multi-scale topology optimization to obtain the optimal combination of macro-layout and local grading for lattice structures based on triply periodic minimal surfaces (TPMSs).

As a porous topology with three-dimensional periodicity and zero mean curvature characteristics, triply periodic minimal surface (TPMS) structures achieve uniform stress transfer through a continuous, uninterrupted surface topology. Their specific stiffness and specific strength are significantly higher than those of traditional honeycomb and truss structures. Moreover, they exhibit a flatter stress plateau under compressive loads, which enables effective absorption of impact energy—a property that endows them with significant application potential in lightweight load-bearing components for aerospace engineering and anti-collision structures in automobiles [42]. The porosity of TPMS structures can be precisely regulated, and their interconnected pore networks can simulate human bone structures. This not only provides sufficient space for cell adhesion and proliferation but also promotes the transport of nutrients and the excretion of metabolic waste. With excellent biocompatibility and osseointegration, TPMS structures are therefore widely used in the biomedical field [43]. The unique pore structure of TPMS structures gives them an extremely high specific surface area. Additionally, when fluid flows through their curved channels, the degree of turbulence is higher, resulting in excellent heat dissipation capacity and a favorable heat transfer coefficient. These features make TPMS structures suitable for efficient heat dissipation in high-power electronic devices.

The limitations of TPMS structures are equally prominent. Firstly, the high-precision forming of complex TPMSs is highly sensitive to additive manufacturing process parameters. Suspended solid parts lack support points, making sintering and forming difficult; when the structural size is small, issues such as surface collapse or internal defects (unfused pores) are prone to occur [44]. Additionally, although TPMS structures exhibit excellent overall mechanical properties, studies have shown that their elastic modulus and compressive strength vary markedly under different loading directions. Particularly in the design of gradient TPMS structures, local topological mutations easily lead to stress concentration. When subjected to multi-directional composite loads, the fatigue life of TPMS structures decreases significantly [45].

The unique geometrical features of TPMS lattice structures also make them highly prone to manufacturing defects during additive manufacturing, thereby impairing the mechanical performance of the fabricated components. First, TPMS architectures contain smooth and continuous surfaces with regions of high curvature, where pronounced local curvature variations tend to increase surface roughness and consequently reduce fatigue strength. Second, the presence of thin-walled regions within TPMS lattices may lead to incomplete melting of the local powder, resulting in pore formation and a subsequent reduction in load-bearing capacity. Moreover, TPMS structures incorporate numerous internal overhanging and inclined surfaces. Unsupported internal overhangs are susceptible to warping during the build process, whereas inclined surfaces with small inclination angles and significant curvature variations are prone to the staircase effect, which degrades surface quality, introduces stress concentrations, and ultimately diminishes fatigue performance.

### 2.4. Design of Biomimetic Lattice Structure

**Figure 3:**
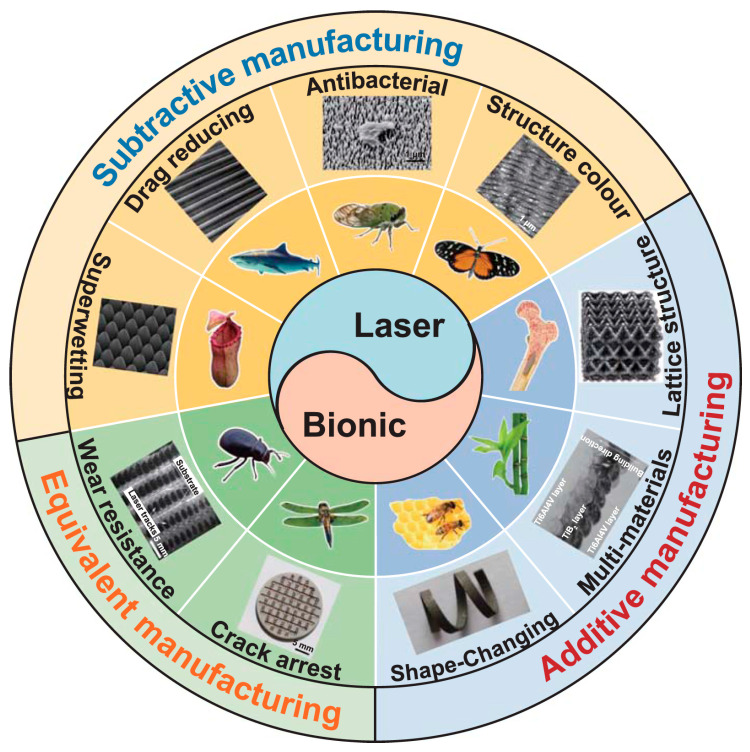
Biomimetic lattice structure. Reprinted from Ref. [52].

After hundreds of millions of years of evolutionary processes, organisms in nature have developed structures adapted to complex environments and exhibit excellent mechanical properties [46,47]. Inspired by this, researchers at home and abroad have designed bionic lattice structures with biological characteristics by studying biological structures in nature that withstand impact loads [48,49,50]. Bionic lattice structures are primarily designed using the topological forms, material distribution patterns, or functional mechanisms of natural biological systems—such as animal bones, plant fibers, insect exoskeletons, and deep-sea sponges—as prototypes. By extracting the core mechanical and functional characteristics of biological structures and combining the porosity and designability of lattice structures, a porous structure system with periodic or quasi-periodic features is constructed [51]. The research team led by Professor Zhang from Jilin University once summarized a variety of bionic lattice structures as shown in Figure 3; it can be seen from the figure that most lattice structures draw inspiration from biological structures in nature.

The core characteristics of bionic lattice structures lie in mimicking lattice-like features found in nature, enhancing the energy absorption capacity of materials, and achieving a balanced design between lightweight properties and high performance. Many structures in nature exhibit excellent energy absorption capacity, such as honeycombs and scales. Doodi and Gunji [53,54] designed bionic circular lattice structures with overlapping areas by mimicking the overlapping structure of fish scales and the hollow circular structure of bamboo; they also designed bionic hexagonal lattice structures by imitating honeycombs and scale structures. Both types of bionic structures possess outstanding energy absorption capacity and are suitable for applications in the automotive and aerospace fields. Excellent energy absorption capacity is a key focus of researchers; however, lightweight design is even more critical in the aerospace field. Insect exoskeletons in nature are excellent lightweight structures, providing valuable insights for lightweight design. For example, inspired by the hyperbolic geometric structure of the outer shell of beetles’ forewings, Meng et al. [55] designed a lightweight and high-strength hyperbolic lattice structure. In addition, mechanical properties are also a key focus of bionic lattice structures. For instance, based on the geometric characteristics of morning glories, Li et al. [56] designed a metamaterial lattice structure with negative Poisson’s ratio; furthermore, Li and Sun [57] designed a three-directional orthogonal lattice structure with excellent compressive performance by mimicking the skeleton of deep-sea glass sponges.

### 2.5. Design of Gradient Lattice Structure

**Figure 4:**
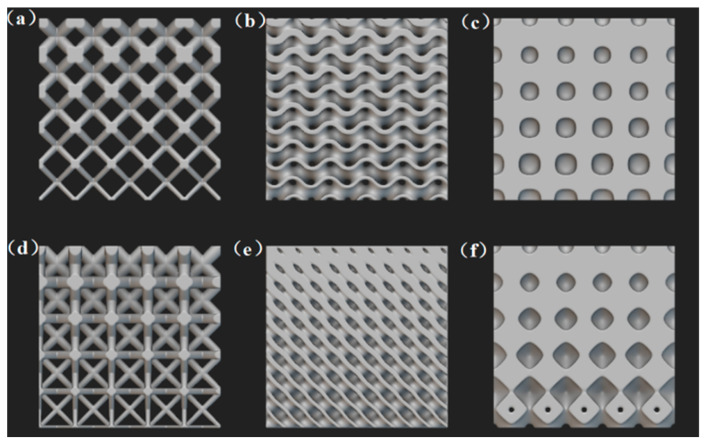
Common gradient lattice structures: (**a**) BCC structure; (**b**) gyroid structure; (**c**) Neovius structure; (**d**) FCC structure; (**e**) diamond structure; (**f**) Schwarz structure.

As shown in Figure 4, are common gradient designs for lattice structures. A gradient lattice structure refers to a non-uniform lattice topology in three-dimensional space, in which one or more unit-cell parameters (such as relative density, rod diameter, pore size, or topology type) are adjusted through mathematical methods or algorithms to produce continuous or stepwise variations along a specific direction or spatial location [58]. Gradient lattice structures can achieve lightweight properties by removing most materials from non-critical regions, serving as an effective approach to enhance the performance of lattice structures. This design can significantly improve the structural mechanical properties and energy absorption performance [59]. In terms of mechanical properties, the continuous variation in relative density or topology type enables precise matching with external load distribution, achieving load-bearing adaptability at different positions [60]. In terms of permeability and heat transfer performance, the gradient variation in density or unit cell type can increase the overall porosity of the structure, thereby enhancing its permeability and heat transfer performance.

Various methods exist for the gradient design of lattice structures. Among them, topology optimization can determine the optimal material distribution within the lattice and enable gradient design while maintaining adequate mechanical strength, making it widely used in the development of gradient lattice structures. For example, Huang et al. [61] conducted an optimal gradient layering design for a lattice structure with the goal of maximizing the lattice stiffness, which significantly improved the load-bearing capacity of the gradient lattice structure. In addition, topology optimization can achieve precise matching between material distribution and load paths, thereby fully enhancing the performance of lattice structures. For instance, based on load paths, Zhao et al. [62] determined the optimal density distribution of the lattice structure through topology optimization and designed a functionally graded lattice structure, whose specific stiffness and specific strength were significantly improved. The essence of gradient lattice structure design lies in grading the parameters of the lattice structure along one or multiple directions. For example, Al-Ketan et al. [63] designed three types of gradient lattice structures with different parameter gradations, namely relative density grading, unit size grading, and multi-morphological (lattice type) grading.

The smaller volume ratio and larger porosity of gradient lattice structures make them suitable not only for lightweight design but also for heat dissipation design. Taking diamond tools as an example, the untimely heat transfer during the machining process of diamond tools will seriously damage their service life. However, gradient lattice structures can reduce the density of non-working areas to improve the overall heat dissipation performance, and their larger porosity can also provide more channels for coolant to pass through. Therefore, gradient lattice structures are applicable to the field of diamond tools with extremely high heat dissipation requirements. Gradient lattice structures possess excellent mechanical properties, heat dissipation performance, and good lightweight characteristics. However, balancing mechanical properties with lightweight design, as well as mechanical properties with heat dissipation performance, imposes extremely high requirements on the design and precision machining of gradient lattice structures, which necessitates further optimization of design technologies and manufacturing processes.

### 2.6. Summary of the Advantages and Disadvantages of Different Types of Lattice Structures

As shown in Table 2, a summary of the advantages and disadvantages of the five lattice types is provided. The comparison evaluates the relative performance of truss, honeycomb, TPMS, biomimetic, and gradient structures in terms of specific strength, energy absorption efficiency, manufacturability, and typical applications.

## 3. Metallic Lattice Structure Additive Manufacturing Process

### 3.1. Powder Bed Fusion (PBF) Process

**Figure 5:**
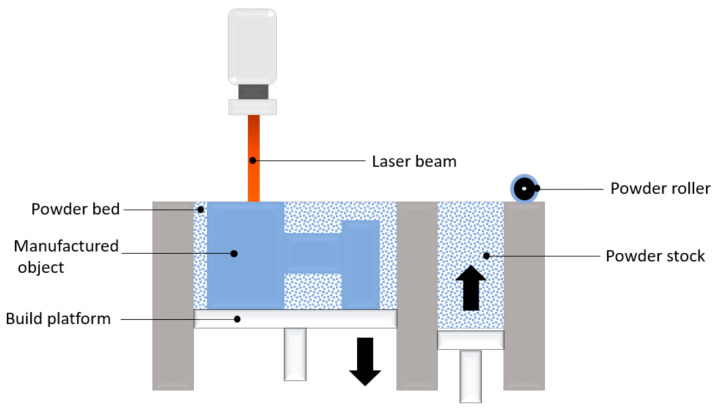
Schematic diagram of PBF process principle. Reprinted from Ref. [64].

Powder Bed Fusion (PBF) is an additive manufacturing (AM) process that uses thermal energy to selectively melt particulate materials into solid shapes. As shown in Figure 5 is the schematic diagram of PBF process principle. A PBF equipment is primarily consists of a powder hopper and a build chamber. A powder recoater transfers the metal powder from the powder hopper to the build chamber and spreads it into a powder bed. Then, based on the information provided by the CAD file, a focused energy source is used to selectively melt specific areas of the powder bed; the powder undergoes physical metallurgy and solidifies into shape under the action of thermal energy, completing the printing of one layer. Subsequently, the powder hopper is incrementally raised and the build chamber correspondingly lowered. This layer-by-layer fabrication cycle is repeated, allowing successive layers to be deposited and consolidated to form the final three-dimensional solid.

Powder Bed Fusion (PBF) technology holds significant advantages in manufacturing lattice structures. PBF technology offers an extremely high degree of design freedom; based on its layer-by-layer deposition forming principle, it can accurately fabricate lattice structures with various complex geometric shapes according to 3D CAD models, and theoretically, it is capable of producing geometric configurations of arbitrary complexity. Among the various PBF technologies, Laser Powder Bed Fusion (L-PBF) achieves notably high manufacturing precision owing to its high-resolution laser processing and controlled layer-by-layer melting mechanism. It can be used to produce Metallic parts with fine microstructures, high density, high strength, and excellent fatigue performance, making it the preferred choice for manufacturing key components of high-performance, high-load-bearing complex structures. Taking Metallic lattice structures as an example, since the manufacturing process pursues extreme precision and performance, and especially when the part size is small, the PBF process is the optimal choice.

### 3.2. Direct Energy Deposition (DED) Process

**Figure 6:**
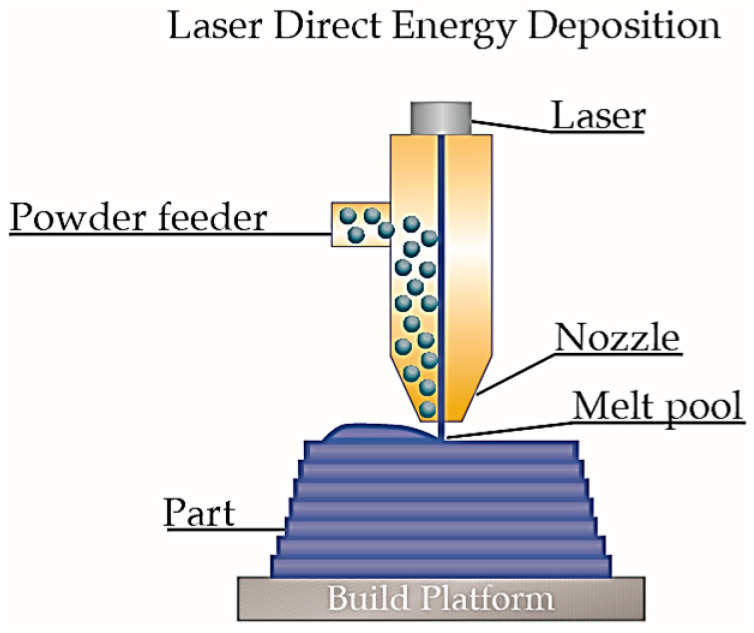
Schematic diagram of DED process principle. Reprinted from Ref. [65].

Directed Energy Deposition (DED) is an additive manufacturing technique that employs a focused energy source—such as a laser, electron beam, or plasma beam—to selectively deposit material layer by layer onto a substrate. Feedstock, typically in powder or wire form, is delivered into a high-temperature melt pool generated by the energy source, where it is melted and subsequently solidified to build a three-dimensional component. As shown in Figure 6 is the schematic diagram of DED process principle: an energy source (a laser beam in the figure) forms a local molten pool on the surface of the substrate or deposited layer; materials (Metallic powder in the figure) are delivered to the local molten pool by a nozzle, carried by a shielding gas (inert gas); upon entering the molten pool, the materials are rapidly melted under the action of the laser beam, and after forming a metallurgical bond with the substrate or the solidified material of the previous layer, they gradually solidify, completing the deposition of a single material track. Subsequently, the aforementioned melting–deposition–solidification process is repeated continuously, and layers are stacked to construct a 3D solid.

In advanced DED systems, the nozzle assembly has three degrees of freedom (X, Y, and Z), while the platform has two degrees of freedom, namely R (rotation) and T (tilt). This five-axis linkage provides an extremely high level of flexibility for the DED process to manufacture parts with complex geometric structures [65]. Owing to the ultra-high flexibility of the nozzle and platform, materials can be delivered to the target surface from any angle, offering a high degree of design freedom. When machining internal cavities and simple lattice structures, no additional supports are required—thus, the DED process is suitable for manufacturing large-scale truss structures. The DED process features a high deposition rate, with a printing speed far exceeding that of powder-spreading additive manufacturing processes such as Powder Bed Fusion (PBF). However, owing to its relatively limited manufacturing precision, the DED process is more suitable for producing large-scale lattice structures with simple geometries, such as strut-based configurations (truss lattice structures) and sandwich structures consisting of panel faces with integrated truss cores. Furthermore, many DED process instruments are equipped with subtractive machining systems, enabling the implementation of a workflow that combines additive manufacturing first followed by subtractive finishing. This integrated approach can significantly improve the surface quality of parts.

### 3.3. Binder Jetting (BJ) Process

**Figure 7:**
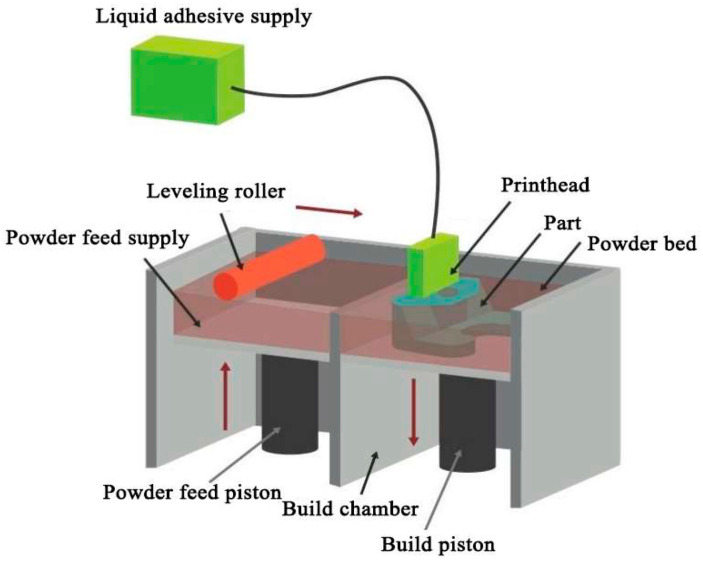
Schematic diagram of BJ process principle. Reprinted from Ref. [66].

Binder Jetting (BJ) is another powder bed-based additive manufacturing technology, primarily utilizing binder jetting to shape powder materials. As shown in Figure 7 is the schematic diagram of BJ process principle; as illustrated in the diagram, the primary distinction between the Binder Jetting process and Powder Bed Fusion (PBF) lies in their powder bonding mechanisms. Unlike PBF, which fuses powder through energy-beam-induced melting, Binder Jetting employs a printhead (as shown in the figure) to selectively deposit a liquid binder onto the powder bed. This bonds the powder in selected areas within a single layer and also adheres it to the previously printed layer, thereby manufacturing a 3D solid layer by layer. Subsequently, similar to the powder spreading process in the PBF process, the powder bed is lowered, and a new layer of powder is spread on top of it. This process is repeated until the solid is fully fabricated.

The Binder Jetting (BJ) process shares many advantages of the Powder Bed Fusion (PBF) process and does not require energy beam equipment. It utilizes powder self-support, eliminating the need for additional support structures and enabling the manufacturing of complex lattice structures. However, precisely due to the absence of energy beam equipment, the Metallic structures produced via BJ require post-processing (debinding and sintering) to meet performance requirements. Owing to this necessary post-processing, the manufacturing precision of the BJ process is difficult to guarantee—it is generally lower than that of the PBF process but higher than that of the Direct Energy Deposition (DED) process. Additionally, the Metallic parts fabricated by the BJ process exhibit relatively poor mechanical properties. However, since the powder does not need to be melted, rapid layer deposition is possible, resulting in a very fast manufacturing speed. Consequently, the BJ process is not well suited for producing metallic components that must withstand high loads, severe impact, or demanding fatigue conditions. However, when low-cost and rapid fabrication of large quantities of metallic lattice structures is required—and when relatively modest performance requirements and subsequent sintering are acceptable—the BJ process represents an optimal manufacturing option.

### 3.4. Wire Arc Additive Manufacturing (WAAM) Process

**Figure 8:**
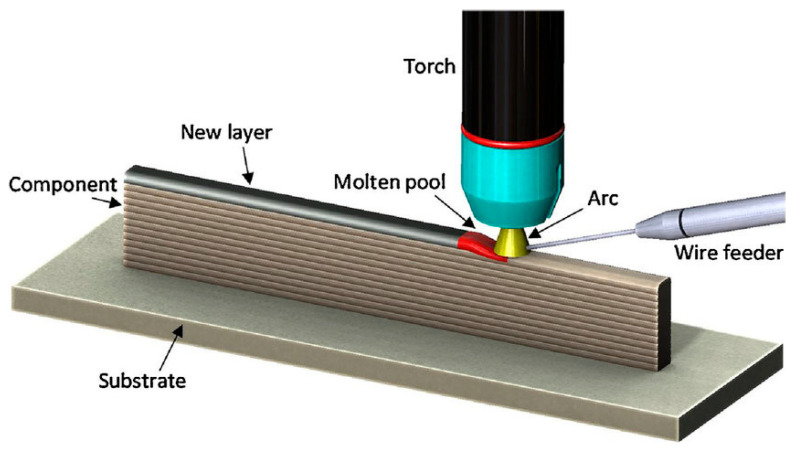
Schematic diagram of WAAM process principle. Reprinted from Ref. [68].

As shown in Figure 8 is the schematic diagram of WAAM process principle. This process employs a welding arc as the heat source to melt metallic wire, depositing each layer onto the substrate following a predefined tool path, and continuing the layer-by-layer welding sequence until the metallic structure is fully formed. Owing to its unique advantages—including high deposition rate, low cost, and the ability to form large-sized components—the Wire Arc Additive Manufacturing (WAAM) process can be used to manufacture large-scale lattice structural parts [67].

However, the Wire Arc Additive Manufacturing (WAAM) process exhibits characteristics such as multi-physical field coupling in layer-by-layer arc deposition, nonlinear time variation, and heat accumulation [67,69], which severely restrict the precision of manufactured components. Components produced via the WAAM process have poor surface quality and cannot meet the requirements for high precision and high surface finish; therefore, post-processing is generally required to treat the component surfaces. Compared with the Powder Bed Fusion (PBF) process and Direct Energy Deposition (DED) process, the WAAM process has the advantages of high forming efficiency and low manufacturing cost, but it also suffers from the drawback of low processing precision. Thus, in the field of Metallic lattice structure manufacturing, the WAAM process is more suitable for fabricating extra-large Metallic lattice structures with simple configurations and low surface quality requirements.

### 3.5. Comparison of the Advantages and Disadvantages of Four Additive Manufacturing (AM) Processes

As shown in Table 3, the advantages and limitations of the four additive manufacturing processes are comprehensively summarized. The comparison offers a systematic evaluation of the performance of PBF, DED, BJ, and WAAM with respect to five critical criteria—feature resolution, surface roughness, build rate, relative cost, and their respective suitability for fabricating different categories of lattice architectures.

## 4. Performance and Application of Metallic Lattice Structure in Additive Manufacturing

### 4.1. Mechanical Properties

Lightweight design holds crucial significance in the aerospace field. The self-weight of spacecraft and aircraft directly affects their fuel efficiency and range; lightweight design can significantly reduce the weight of aerial vehicles, thereby lowering fuel consumption and improving flight speed and maneuverability. As a structural-functional integrated material with high specific strength and specific stiffness, Metallic lattice structures can reduce structural weight while maintaining excellent mechanical properties. They serve as core carriers for lightweight design and can meet the dual requirements of aerospace equipment for lightweight performance and load-bearing capacity [70]. For example, Zhang et al. [71] designed a lattice structure applicable to aircraft compressor impellers. This lattice structure not only satisfies the lightweight and mechanical performance requirements of compressor impellers and enhances overall compressor efficiency, but also allows for the tuning of impeller stiffness and strength through adjustments to the lattice parameters. The application of lattice structures in aircraft compressor impeller structures can not only reduce the overall weight of the aircraft but also decrease the torque generated when the impeller starts and stops. Among the numerous components of an aircraft, the wing structure has always been regarded as one of the most critical components due to its high stress requirements. Moreover, the wing acts as a cantilever beam and serves as a primary load-bearing structure [72]. For example, Khan et al. [73] have experimentally verified the feasibility of replacing traditional aircraft wings with lattice-filled wings. In particular, wings filled with Kelvin lattice structures exhibit smaller wingtip deflection, lower weight, and lower stress, making them an excellent choice for lightweight wing structures. This type of lightweight material—characterized by internal lattice fillers and an enclosed outer shell—has also attracted significant attention in the field of aerospace satellites. Boschetto et al. [74] designed a satellite structure with enclosed lattices and further implemented a thickening design for the critical regions of the lattice structure. The newly designed lattice satellite structure not only reduces weight but also meets the requirements for mechanical performance. The continuous advancement of additive manufacturing technology will promote the widespread application of lattice structures in the aerospace field. This technology enables the fabrication of complex lattice structures that are challenging or impossible to manufacture using conventional methods, thereby offering expanded possibilities for lightweight design.

Negative Poisson’s ratio materials are a type of novel metamaterials with auxetic behavior. When these materials undergo tensile deformation in the longitudinal direction, their transverse dimensions increase accordingly, and vice versa [75]. Owing to their unique mechanical and physical properties, negative Poisson’s ratio materials can enhance in-plane indentation resistance and transverse shear modulus, while also improving fracture toughness, dynamic performance, and thermal shock strength [75,76]. Lattice structures possess special geometric configurations, enabling them to exhibit the negative Poisson’s ratio effect. Lattice-structured metamaterials exhibiting negative Poisson’s ratio behavior undergo inward contraction and localized strain concentration under compressive loading, thereby enhancing the performance of tactile sensors. For example, based on the negative Poisson’s ratio characteristics of cubic lattices with spherical voids, Kang et al. [77] designed two types of sensors: a capacitive sensor that responds to pressure by adjusting electrode spacing and dielectric distribution, and a resistive sensor based on a conformal coating network of carbon nanotubes. Tests have demonstrated that compared with traditional porous geometric structure sensors with positive Poisson’s ratio, the negative Poisson’s ratio sensors exhibit higher sensitivity. Lattice structures with negative Poisson’s ratio properties have attracted significant attention. In particular, 3D negative Poisson’s ratio metallic metamaterial lattice structures, due to their more superior performance, have been the subject of research by an increasing number of scholars. For example, Li et al. [78] from Guangzhou University designed and improved a 3D negative Poisson’s ratio metallic metamaterial lattice structure by adding star-shaped structures to the traditional 3D concave structure, which significantly enhanced the structural stability and stiffness. Additionally, Gao et al. [79] designed a negative Poisson’s ratio anti-chiral 3D polyhedral lattice structure. Studies have shown that this structure exhibits higher fracture energy and can absorb more energy during the fracture process.

### 4.2. Energy Absorption Performance

Lattice structures exhibit excellent energy absorption capacity through the synergy between periodic cell topology design and material mechanical behavior [80], and can efficiently dissipate external impact energy via controllable plastic deformation, buckling, or fracture processes. They have wide-ranging applications in fields with stringent requirements for energy absorption performance, such as the aerospace and automotive industries.

In the aerospace field, aircraft, satellites, and spacecraft operate in complex environments, imposing extremely high requirements on the energy absorption and protection performance of their structures. Taking aircraft as an example, when they collide with foreign objects such as birds during high-speed flight, severe damage to the aircraft body may occur. However, lattice structures can effectively absorb impact energy through their inherent deformation and failure mechanisms, thereby reducing the damage transmitted to the internal components of the aircraft body during a collision. Alvian et al. [81] compared the specific energy absorption capacities of various strut-based lattice structures. Their study revealed that the Octet lattice structure is the optimal lattice configuration for crashworthy components. Furthermore, they applied the distorted Octet lattice structure to the surface structure of the aircraft undercarriage; this distorted lattice structure can effectively absorb the impact energy generated when the aircraft lands. This proves that lattice structures can be well applied in crashworthy components of aircraft. Lattice structures, which possess excellent strength-to-weight ratios and energy absorption capabilities, have been widely used as protective layers in various fields, including aircraft fuselage protective layers, satellite and spacecraft protective layers, and automotive body protective layers. To optimize the performance of lattice structure protective layers, Professor Zhou’s research team [82] from Nanyang Technological University, Singapore, designed a layered lattice structure composed of alternating Face-Centered Cubic (FCC) and Body-Centered Cubic (BCC) lattice structures. The specific energy absorption and energy absorption efficiency of this new layered structure are more than 10 times and 9 times higher than those of the BCC structure, respectively. Furthermore, it can provide dual mechanical protection for both the internal and external parts of the structure. Satellites and aerospace reentry capsules typically have small volumes, compact structures, and must withstand large impact forces during landing; therefore, their energy absorption capacity is particularly critical. Addressing this issue, Wang et al. [83] proposed the use of truss lattice-filled sandwich structures, which can achieve lightweight design of aerospace reentry capsules and effective absorption of landing impact energy.

Moreover, large-scale space stations in the aerospace sector impose even more stringent demands on structural lightweighting and energy absorption capacity, and lattice structures are likewise well suited to meet these requirements. For example, Professor Xiong’s research team from Harbin Institute of Technology [84] designed a modularly assembled lattice structure. This structure possesses excellent mechanical properties and energy absorption capacity, enabling its application in large-scale aerospace space structures such as lunar bases, space telescopes, and space power stations. Metallic lattice structures, relying on their superior energy absorption capacity and lightweight characteristics, have been widely used in structural design within the aerospace field. Furthermore, through ongoing optimization and design refinement, these structures can be tailored to satisfy increasingly stringent and diverse requirements for protection and energy absorption.

The excellent energy absorption performance of metallic lattice structures also finds wide application in the automotive field, such as in automotive energy absorbers and anti-collision beams. Zhang et al. [85] found through their research that energy absorbers with plate cubic lattice structures exhibit outstanding impact resistance and energy absorption capacity, which can meet the dual requirements of automotive lightweighting and protection. Liang et al. [86] from Jilin University conducted a comparative analysis of the crashworthiness of lattice structure-filled energy absorbers and traditional energy absorbers under multi-angle oblique collision conditions. Their study revealed that, compared with traditional automotive energy absorbers, automotive energy absorbers filled with regular hexagonal lattice structures not only have stable and excellent energy absorption performance but also their mass is significantly lower than that of traditional ones. The application of lattice structures in automotive energy absorbers can, on the one hand, enhance the energy absorption capacity of the absorbers and, on the other hand, reduce the weight of the automotive body, thereby meeting the lightweight requirements of automobiles. In the field of anti-collision beams, when metallic lattice structure-based anti-collision beams are subjected to collision impacts, their unit cells achieve stable dissipation of kinetic energy through ordered layer-by-layer collapse, plastic buckling, or local shear deformation. This avoids the sudden fracture of traditional solid or hollow anti-collision beams caused by stress concentration; thus, anti-collision beams with metallic lattice structures possess better structural stability and protective capability. Wu et al. [87] designed a gradient lattice structure with negative Poisson’s ratio characteristics and applied it to automotive anti-collision beams. Their study revealed that this lattice-structured anti-collision beam not only has better energy absorption capacity but also can reduce harm to pedestrians, providing improved pedestrian protection. Lattice-structured anti-collision beams not only offer superior protective performance compared to traditional solid anti-collision beams but also have a lighter mass. Rajpura et al. [88] found through their research that automotive anti-collision beams with Octet lattice structures not only exhibit better energy absorption capacity than traditional solid lattice-structured anti-collision beams but also have a significantly reduced weight. Triply Periodic Minimal Surface (TPMS) lattice structures possess better collision energy absorption capacity than other lattice structures. By comparing the energy absorption capacities of various types of TPMS structures with different parameters, N. D. Cresswell et al. [89] found that the gyroid-type TPMS structure with a unit cell size of 10 mm and a surface thickness of 2 mm has the optimal specific energy absorption capacity (13.06 J/g (±0.15)), which is significantly superior to the 3D truss structures and traditional 2D lattice structures currently used in the automotive industry. In conclusion, the application of metallic lattice structures in automotive anti-collision structures can not only enhance energy absorption capacity to better protect the vehicle body and pedestrians but also reduce the vehicle body weight to achieve lightweight design and lower automobile fuel consumption.

### 4.3. Thermal Properties

Lattice structures exhibit excellent flow and heat transfer performance due to their characteristics such as smooth surfaces, periodically connected pores, and high specific surface area. The high-density heat dissipation channels inside lattice structures can significantly enhance heat dissipation performance; meanwhile, they allow the flow of cooling fluid to achieve active heat reduction. Under forced convection conditions, heat conduction through solid struts becomes the main heat dissipation mechanism. The complex pore structure of lattice structures can disrupt fluid flow, strengthen heat conduction between the wall surface and struts, and thereby improve heat exchange efficiency [90]. Consequently, lattice structures are widely used in heat sink design owing to their superior heat dissipation performance [91]. The high porosity of lattice structures can significantly reduce the continuity of solid materials, leading to a substantial extension of heat conduction paths. Furthermore, by introducing thermal barriers within the pores, heat transfer can be effectively suppressed. As a result, lattice structures also exhibit excellent thermal insulation performance.

Metallic lattice structures are widely used in the heat dissipation field due to their excellent heat dissipation performance. Taking micro-satellites as an example, their internal equipment is complex and compact, imposing strict requirements on heat dissipation systems. Chen et al. [92] proposed a cooling system composed of a hollow metallic microlattice structure filled with liquid. Their study demonstrated that this novel lattice-structured cooling system can satisfy the heat dissipation requirements of most microsatellite architectures while maintaining exceptionally low structural weight. The complex internal channels of lattice structures allow the introduction of cooling liquid and provide turbulent flow conditions, thereby enhancing the heat dissipation effect of lattice-structured heat sinks. With the rapid development of electronic technology, electronic devices are increasingly moving toward miniaturization and integration, which makes heat dissipation for electronic devices even more critical. Additive manufacturing technology enables the precision fabrication of tiny and complex lattice-structured heat sinks, producing lattice-structured heat sinks for electronic devices with excellent heat dissipation performance.

Lattice structures are also excellent structural choices for heat exchangers. In particular, for TPMS structures, the internally complex interconnected pores endow them with an extremely high surface area. By expanding the contact area between the fluid and the solid, they can significantly enhance the convective heat transfer coefficient. Kilic [93] found through research that the temperature uniformity of the silver (Ag) TPMS structure with nanofluid added is 40% higher than that with pure water added; the combination of the silver (Ag) TPMS structure and nanofluid can significantly improve cooling performance. A major advantage of lattice-structured heat exchangers is that they can adapt to different cooling requirements by replacing the cooling fluid in the internal channels. Another significant advantage of lattice-structured heat exchangers is that they possess excellent heat transfer performance while maintaining good mechanical properties. Kevin J. Maloney et al. [94] have proven through research that microlattice heat exchangers have versatile capabilities in heat transfer, load-bearing, and energy absorption. Therefore, multifunctional heat exchangers with lattice structures can be applied in heat transfer scenarios where size and weight are restricted, including those in the automotive, aerospace, and aviation fields.

Lattice structures possess excellent thermal insulation performance and thermal protection capabilities. Particularly in the aerospace field, aircraft experience intense friction with the atmosphere during high-speed flight, leading to a sharp increase in surface temperature, which poses a severe threat to the structural safety of the aircraft. Owing to their excellent thermal insulation performance and lightweight characteristics, metallic lattice structures have become ideal materials for aircraft thermal protection systems. For example, Wang et al. [95] designed a lattice structure with a large aspect ratio. Compared with conventional lattice structures, this large-aspect-ratio lattice structure achieves superior thermal insulation performance. Moreover, the large-aspect-ratio lattice still maintains high thermal insulation efficiency even after large deformation or fracture, making it well-suited for thermal protection of aircraft. Similarly, Craig A. Steeves et al. [96] designed a multifunctional thermal protection structure for aircraft using zero-expansion lattices and verified the feasibility of this structure. With their excellent thermal insulation performance and thermal protection capabilities, lattice structures can be well applied in the aerospace field. They not only fulfill the function of thermal protection but also meet the requirement of lightweight design.

### 4.4. Biocompatibility

Lattice structures can, through the synergy of topological design and material properties, precisely match the structural and functional requirements of biological tissues. Consequently, they demonstrate excellent biocompatibility and possess significant potential for applications in the biomedical field. For instance, in the case of human bone structures, metallic lattice structures can be tailored to closely match the mechanical properties of natural bone by adjusting structural parameters and optimizing topology. Furthermore, the porous nature of these lattice structures offers an ideal environment for bone tissue regeneration, thereby facilitating the recovery of bone structures.

In the field of bone repair and replacement, lattice structures can be tailored to match the properties of human bones by adjusting parameters, making them excellent human bone implants. Rezapourian et al. [97] developed a novel Split-P TPMS lattice as the base structure; by regulating its curvature, they adjusted the lattice’s elastic modulus and compressive strength to values close to those of cancellous bone and cortical bone, making it suitable for load-bearing bone implants. Adjusting the porosity of lattice structures not only enables their mechanical properties to approximate those of human bones but also enhances the adhesion rate of osteocytes. Alaña et al. [98] simulated the mechanical anisotropy of bone tissue by adjusting parameters. Their study revealed that when the porosity was 70%, the elastic modulus of the lattice was highly matched with that of cancellous bone, and the adhesion rate of osteoblasts increased by 40%—making it suitable for load-sensitive implants such as spinal fusion devices. Lattice structures possess outstanding and highly tunable mechanical properties, enabling them to closely match the mechanical characteristics of bone tissue at the implantation site. Moreover, they exhibit strong osteocyte adhesion and excellent biocompatibility.

Bone scaffolds are an effective approach for treating bone defects, as they can provide mechanical support to the bone defect site and restore its mechanical properties [99]. TPMS bone scaffolds have emerged as one of the popular choices for bone defect repair due to their unique biological properties, such as permeability, specific surface area, and biocompatibility. The pore structure based on Triply Periodic Minimal Surfaces (TPMSs) is conducive to optimizing the performance of bone scaffolds, enhancing their biocompatibility and mechanical properties, and promoting the regeneration and repair of bone tissue. TPMS structures possess high porosity, which can provide more space for intracellular growth and the formation of new blood vessels. This facilitates cell migration, proliferation, and differentiation, and improves the bioactivity and biocompatibility between the bone scaffold and surrounding tissues [100]. Jing et al. [101] designed a metallic porous bone scaffold based on the TPMS structure and infiltrated a gelatin-alginate-magnesium-lithium silicate (GE-SA-LAP) composite polymer into the metallic scaffold, fabricating a structurally bionic functional composite bone scaffold. The TPMS structure can mimic the microstructure and mechanical properties of natural trabecular bone, while the GE-SA-LAP composite polymer can simulate the extracellular matrix (ECM) microenvironment, optimizing the conditions for osteocyte attachment and proliferation. This GE-SA-LAP composite metallic bone scaffold based on the TPMS structure not only meets the mechanical property requirements of bone tissue but also exhibits excellent biocompatibility. It provides an ideal biological microenvironment for bone tissue cells, which is highly beneficial for bone tissue repair.

In addition to their applications in bone therapy, lattice structures can also be utilized as cardiovascular stents. Traditional cardiovascular stents often struggle to conform to the diverse geometries of blood vessels, such as the curved and bifurcated segments of the cardiovascular system. However, microlattice structures, produced through additive manufacturing, allow for the personalized design of biomedical devices. Cardiovascular stents require both optimal permeability and adequate radial support, making lattice structures an ideal solution for this purpose. For example, Xiao et al. [102] designed and fabricated a thin-walled vascular stent with a negative Poisson’s ratio microlattice structure. Through radial reinforcement, they reduced the stent thickness by 10–20% while increasing its radial compression resistance by 70%. Additionally, this stent exhibits excellent cytocompatibility.

## 5. Summary

### 5.1. Summary of Research Status

In recent years, with the rapid development of additive manufacturing technology, additively manufactured metallic lattice structures have been widely applied. This paper systematically reviews the classification, design, processes, and applications of additively manufactured metallic lattice structures. Currently, significant progress has been achieved in research related to additively manufactured metallic lattice structures.

In terms of lattice structure classification, different types of structures exhibit distinct properties. Truss lattice structures can achieve significant improvements in mechanical performance through adjustments to strut parameters and node optimization. However, they inherently suffer from stress concentration at the nodes, which remains a critical limitation. Honeycomb lattice structures enhance the negative Poisson’s ratio effect and energy absorption capacity through structural optimization and innovation, thereby improving stability under dynamic loads. Triply Periodic Minimal Surfaces (TPMSs) are controlled by implicit function equations and feature complex interconnected internal channels. While possessing high specific strength and specific stiffness, they also exhibit characteristics of high specific surface area and high porosity, endowing them with excellent mechanical properties, thermal properties, and permeability. Bionic lattice structures realize the synergistic optimization of structure and function by mimicking biological structures in nature, breaking through the performance limitations of inherent configurations. Gradient lattice structures achieve gradient variations in various properties through changes in unit cell type, size, and density, enhancing the adaptability and stability of the structure to complex working environments.

In terms of additive manufacturing processes, the Powder Bed Fusion (PBF) technique offers high manufacturing precision, but is associated with lower efficiency and higher costs. It is particularly suitable for producing small, complex metallic lattice structures, where the resulting parts exhibit superior performance. The Directed Energy Deposition (DED) process has high manufacturing efficiency but relatively low precision and high cost; it is suitable for processing large-sized and simple metallic lattice structures, and the manufactured parts show good performance. The Binder Jetting (BJ) process features high manufacturing efficiency, relatively low cost for mass production, and moderate manufacturing precision. It is suitable for mass-producing complex parts (which require debinding and sintering processes), but the manufactured parts have relatively poor performance. The Wire Arc Additive Manufacturing (WAAM) process is similar to welding technology; it has high manufacturing efficiency and low cost but poor manufacturing precision and surface quality, requiring post-processing such as machining. Therefore, it is only applicable to processing extra-large-sized metallic lattice structures with simple configurations.

In terms of performance and applications, metallic lattice structures exhibit excellent mechanical properties. Their high mechanical strength and lightweight characteristics make them ideal structures for lightweight design. They possess a progressive deformation mechanism and demonstrate efficient energy dissipation capacity under both quasi-static and dynamic loads, resulting in excellent energy absorption performance. Their advantages of high porosity, high specific surface area, and high connectivity endow them with excellent heat dissipation, heat transfer, and thermal insulation capabilities. Through parameter optimization, they can precisely match the structural and functional requirements of biological tissues, exhibiting excellent biocompatibility and thus being widely applied in the field of bone repair.

### 5.2. Development Trend Outlook

The performance potential of traditional truss, honeycomb, and surface-based lattice structures is inherently limited. To meet the high-performance demands of lattice structures for advanced equipment, novel design approaches such as hybridization, biomimicry, gradient design, and topology optimization are emerging as key research directions. While optimizing properties like lightweight design, energy absorption, and heat dissipation-thermal insulation, certain mechanical properties are inevitably compromised. As a result, the development of strategies to synergistically enhance the overall performance of lattice structures has become an increasingly critical focus.

Additive manufacturing technology has overcome the constraints of traditional manufacturing methods, providing metallic lattice structures with an exceptional degree of design freedom. However, the manufacturing defects inherent in current metallic additive manufacturing processes are significant factors influencing the mechanical properties of lattice structures. Therefore, controlling the microstructure and defects in metallic additive manufacturing may prove to be more critical than the configuration design of the lattice structures themselves.

The surface quality of additively manufactured metallic lattice structures is another issue worthy of significant attention. Even with laser powder bed fusion technology—which achieves the best surface forming quality—the problem of surface roughness in lattice structures still persists. Therefore, an important development direction is to improve the surface quality of metallic lattice structures through post-processing technologies, such as fluid polishing technology.

In summary, the performance enhancement of additively manufactured metallic lattice structures needs to be considered from multiple aspects, including innovative configuration design and manufacturing quality control.

## Figures and Tables

**Table 1:** Typical TPMS structure and its implicit function equation.

TPMS	Entity Class	Thin-Walled Class	Implicit Function Equation
Gyroid	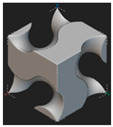	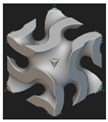	sin x ∗ cos y + sin y ∗ cos z + sin z ∗ cos x = c
Schwarz	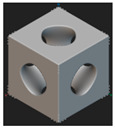	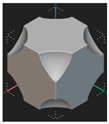	cos x + cos y + cos z = c
Diamond	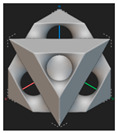	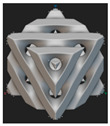	sin x ∗ sin y ∗ sin z + sin x ∗ cos y * cos z + cos x ∗ sin y ∗ cos z + cos x ∗ cos y ∗ sin z = c

**Table 2:** Comparison of advantages and disadvantages of different types of lattice structures.

Structure Type	Specific Strength	Energy Absorption Efficiency	Manufacturability	Typical Applications
**Truss Lattice Structures**	High specific strength; vulnerable to stress concentration at joints.	Moderate energy absorption efficiency.	Good manufacturability.	Lightweight aerospace components, heat exchangers, orthopedic implants (when optimized), mechanical metamaterials.
**Honeycomb Lattice Structures**	High in-plane specific strength; lower out-of-plane strength.	High in-plane energy absorption.	Good manufacturability.	Aerospace sandwich panels, protective packaging, automotive crash absorbers.
**TPMS Structures**	High and uniform specific strength.	Excellent energy absorption efficiency.	The structure is com-plex, requiring high precision from additive manufacturing technology.	Biomedical implants (porous scaffolds), thermal management, acoustic damping, multifunctional metamaterials.
**Biomimetic Lattice Structures**	High specific strength can be achieved by mimicking natural structures.	By mimicking natural structures, high energy absorption efficiency can be achieved.	The structure is more complex, requiring more stringent requirements for the additive manufacturing process.	Bone-mimicking scaffolds, impact-resistant structures, architected materials with superior stiffness-to-weight ratios.
**Gradient Lattice Structures**	Strength can be optimized by spatially varying density or topology.	Very high; gradients promote sequential crushing and superior energy absorption.	The structure is more complex, requiring more stringent requirements for the additive manufacturing process.	Functionally graded implants, heat exchangers, crashworthy structures with tailored deformation behavior.

**Table 3:** Summary of the advantages and disadvantages of four Additive Manufacturing (AM) processes.

AM Processes	Feature Resolution	Surface Roughness	Build Rate	Relative Cost	Suitability for Different Lattice Types
PBF	Very high: best for fine and intricate features	Moderate to rough	Low	High	TPMS; Complex truss lattices and honeycomb lattice; biomimetic structures; Gradient lattices
DED	Moderate: limited by melt pool size	Rough	High	High	large-scale lattices
BJ	Moderate to high: depending on powder size	Moderate to good	Very high	Moderate	TPMS; Honeycomb lattice
WAAM	Low: large bead width limits fine features	Very rough	Very high	Low	large-scale lattice structures
